# OA19 Acute confusion, reduced conscious levels and stroke in systemic lupus erythematosus resulting from cerebral vasculitis

**DOI:** 10.1093/rap/rkae117.019

**Published:** 2024-11-05

**Authors:** Eman Elfar, Christopher Baldwin, Gagandeep Sukhija, Jamie Thoroughgood, Hadi Rabee, Andrew Rutherford, Arti Mahto, Chris Wincup

**Affiliations:** King’s College Hospital, London, United Kingdom; King’s College Hospital, London, United Kingdom; King’s College Hospital, London, United Kingdom; King’s College Hospital, London, United Kingdom; King’s College Hospital, London, United Kingdom; King’s College Hospital, London, United Kingdom; King’s College Hospital, London, United Kingdom; King’s College Hospital, London, United Kingdom

## Abstract

**Introduction:**

Systemic lupus erythematosus (SLE) presents substantial challenges, particularly in cases of major organ involvement, particularly within the central nervous system, which may be characterised by acute neurological symptoms including seizures and altered levels of consciousness. These cases often necessitate careful consideration of potential alternate causes, particularly infection. Brain imaging can often be normal in neurological manifestations of the disease but a wide array of abnormalities have previously been reported on scans. This case underscores the challenges clinicians face in managing complex lupus with severe neurological manifestations, emphasizing the critical importance of promptly recognizing serious symptoms and initiating effective definitive treatment.

**Case description:**

A 20-year-old woman with SLE presented to hospital having been diagnosed with the disease nine months earlier whilst living in the Caribbean. Diagnosis was based on a malar rash, inflammatory arthritis, and peripheral oedema with an active urinary sediment for which she was presumed to have lupus nephritis (LN). She gave a history of one previous episode of generalised tonic clonic seizure, for which she was treated with levetiracetam. At that time, the underlying aetiology of her seizure was unclear. Initial management was with a combination of prednisolone and azathioprine. Upon arrival in the UK, she was hospitalized with worsening peripheral oedema, hypoalbuminemia, and acute kidney injury. A renal biopsy subsequently confirmed mixed Class IV / V LN with active and chronic lesions. In turn she was treated with high-dose glucocorticoids and Azathioprine was switched to Mycophenolate. Despite treatment, her renal function deteriorated to established CKD stage 4.

Shortly after discharge, she presented acutely unwell with diarrhoea, vomiting, hyperkalaemia, an acute on chronic kidney injury and fluid overload. She very acutely dropped her GCS with a CT head showing changes in the left frontal lobe, and an MRI revealed numerous ill-defined lesions suggesting a multiphasic inflammatory process. Despite treatment for possible CNS infection, her condition deteriorated, requiring ICU admission for ventilatory and renal support.

There was uncertainty surrounding brain imaging which further complicated decisions regarding treatment plan. CSF samples and a negative infection screen excluded infections. Initial scans suggested multifocal infarctions, which could be embolic secondary to Libman-Sacks endocarditis. CT angiogram showed extensive generalised vasospasm, supporting a diagnosis of cerebral vasculitis. Intravenous methylprednisolone was started for presumed neuropsychiatric (NP)-SLE. She was treated with cyclophosphamide and rituximab, leading to significant improvement. Her GCS improved to 15/15, and although she remains dialysis-dependent, significant neurological recovery was observed within one month of presentation.

**Discussion:**

Prompt and definitive treatment in NP-SLE is paramount, particularly when addressing acute manifestations presenting with altered conscious levels and seizures. In this case, a multidisciplinary approach enabled the timely identification and treatment and highlighted the benefits of coordinated care among specialists, which is key to improving patient outcomes in severe cases of SLE.

SLE can present with a wide array of acute neurological manifestations, including seizures, psychosis, acute confusion and stroke. This necessitates that all members of the medical team are vigilant and prepared to escalate care rapidly in the context of acute neurological deterioration. The rapid decline in both renal function and neurological status in this patient highlighted the need for immediate and aggressive management, emphasizing the role of high-dose steroids in controlling disease activity with combined therapy with both rituximab and cyclophosphamide concurrently.

A key point in managing such complex cases is the exclusion of infections, which can mimic or exacerbate lupus flares. Despite initial treatment for possible meningoencephalitis, thorough prompt investigation with CSF samples and infection screens it was possible to rule out infectious causes, thus avoiding unnecessary delays in initiating appropriate immunosuppressive therapy.

The rapid initiation of therapy with IV methylprednisolone followed by high-dose oral prednisolone, and subsequently cyclophosphamide and rituximab, demonstrated the effectiveness of aggressive immunosuppressive therapy in achieving significant clinical improvement. Within only a few weeks of starting treatment, the patient made a significant neurological recovery. Her cognitive function including both short and long-term memory was persevered and physically she has rehabilitated well in spite of extensive areas of infarction on cerebral imaging.

Importantly this case underscores that in acute life or organ threatening presentations prompt definitive treatment is warranted when there is strong evidence of an underlying active inflammatory process even in the absence of conclusive diagnostic certainty of the underlying aetiology.

**Key learning points:**

• Prompt and definitive treatment in SLE is crucial to prevent irreversible damage and improve outcomes.

• Early recognition and intervention are essential for managing acute NP-SLE manifestations.

• A multidisciplinary approach is beneficial for timely identification and treatment, involving acute medicine physicians, nephrologists, neurologists, rheumatologists, and intensivists.

• Excluding infections is vital, as they can mimic or exacerbate lupus flares.

• Thorough investigation with CSF samples and infection screens is essential to rule out infectious causes and avoid delays in appropriate treatment.

• Aggressive immunosuppressive therapy, including GC, cyclophosphamide, and rituximab, can lead to significant clinical improvement before the onset of neurological damage.

• Stroke is a well-recognised, although rarely observed, manifestation of NP-SLE that carries significant risk of morbidity and mortality.

